# Management of the refractory vitiligo patient: current therapeutic strategies and future options

**DOI:** 10.3389/fimmu.2023.1294919

**Published:** 2024-01-04

**Authors:** Xinju Wang, Wei Wu, Jianru Chen, Chunying Li, Shuli Li

**Affiliations:** Department of Dermatology, Xijing Hospital, Fourth Military Medical University, Xi’an, China

**Keywords:** refractory vitiligo, treatment, surgery, fractional laser, regenerative medicine, psychotherapy

## Abstract

Vitiligo is an autoimmune disease that leads to disfiguring depigmented lesions of skin and mucosa. Although effective treatments are available for vitiligo, there are still some patients with poor responses to conventional treatment. Refractory vitiligo lesions are mostly located on exposed sites such as acral sites and lips, leading to significant life stress. Understanding the causes of refractory vitiligo and developing targeted treatments are essential to enhance vitiligo outcomes. In this review, we summarized recent treatment approaches and some potential methods for refractory vitiligo. Janus kinase inhibitors have shown efficacy in refractory vitiligo. A variety of surgical interventions and fractional carbon dioxide laser have been widely applied to combination therapies. Furthermore, melanocyte regeneration and activation therapies are potentially effective strategies. Patients with refractory vitiligo should be referred to psychological monitoring and interventions to reduce the potential pathogenic effects of chronic stress. Finally, methods for depigmentation and camouflage may be beneficial in achieving uniform skin color and improved quality of life. Our ultimate focus is to provide alternative options for refractory vitiligo and to bring inspiration to future research.

## 1 Introduction

Vitiligo is a chronic depigmenting disease resulting from disordered immunity and the loss of functional melanocytes (1). The clinical presentation is mainly the presence of depigmented skin and mucosa. As a disfiguring disease, vitiligo affects about 0.5% to 2% of the world’s population without gender bias (2, 3). Whether or not the lesions occur on exposed parts of the skin, vitiligo patients usually have cosmetic anxiety and reduced quality of life (QoL). So far, autoimmunity theory, melanocyte adhesion theory, somatic mosaicism theory, neural theory, microvascular theory, and genetics have all been proposed to explain vitiligo pathogenesis (4). Among the numerous established theories, autoimmunity is the most popular one. It is generally recognized that the aberrantly enhanced innate immunity combined with adaptive immunity promotes the targeted killing of melanocytes by CD8^+^ cytotoxic T-lymphocytes (5, 6). Since the pathogenesis is not entirely clear, there is no single magic bullet in vitiligo management.

Based on the understanding of the mechanism, vitiligo has two main therapeutic goals, one is to prevent disease progression by controlling the abnormal inflammatory state; the second is to induce re-pigmentation by improving the quantity and function of melanocytes in the lesions (7, 8). Vitiligo patients exist in the condition of progressive or stable periods, and there are different treatments for different periods. Thus far, first-line therapy for patients with stable vitiligo usually consists of narrow-band UVB (NB-UVB), topical/systemic corticosteroids, and calcineurin inhibitor therapies (9). Steroid oral minipulse therapy is usually recommended for patients with rapid progression (10). As a Janus kinase (JAK) 1/JAK2 inhibitor, ruxolitinib has passed the clinical phase III trial and has already been approved by the Food and Drug Administration for the treatment of nonsegmental vitiligo (11). While many patients are adequately treated with conventional therapies, a subset of vitiligo patients fail to respond to these treatments and remain refractory.

**Figure 1:**
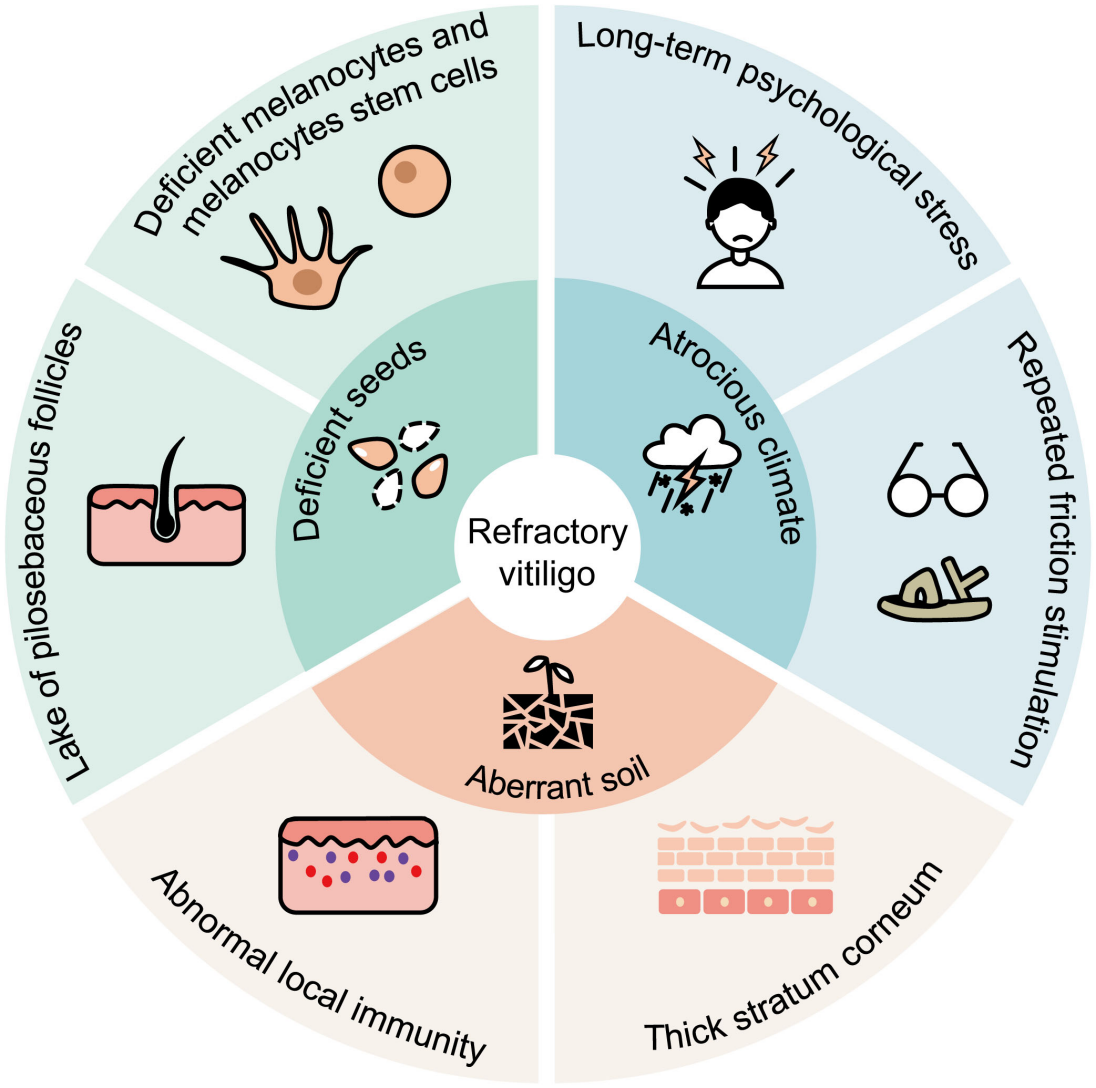
Clinical images depict a vitiligo patient before and after 24-weeks of continuous NB-UVB treatment, followed by recurrence at the original site after stopping treatment.

There is no consensus definition of “refractory” vitiligo, but the term implies the persistence of the disease and resistance to medication/phototherapy (12). Refractory vitiligo has some anatomical location preferences, such as acral and joint sites, bony prominences, and lip (13, 14). There are several potential reasons for the preference of these locations. Most popular theories hold that refractory lesions have fewer pilosebaceous follicles and perilesional melanocytes (15). In addition, these low-response areas have a greater susceptibility to repeated friction and traumatic stimulation. Due to the tissue structure and repeated friction experience, lesions of refractory vitiligo have relatively thicker stratum corneum, which likely affects the penetration of topical drugs and phototherapy (16). Moreover, lesions with white hair are more difficult to get re-pigmentation, since white hair follicles have a lower percentage of intact melanocyte reservoirs than black hair follicles (17). White hair is also one of the characteristics of segmental vitiligo. Segmental vitiligo progresses rapidly and usually damages the follicular melanocyte reservoirs at the early stage (18). This may explain why segmental vitiligo is less responsive to conventional treatment. Psychological stressors are also considered as potential vitiligo triggers (19). The possible causes of refractory vitiligo are summarized in **Figure 1**. Firstly, the absence of pilosebaceous follicles and melanocyte stem cells/melanocytes at the lesions resembles scarce “seeds” for melanin production. Secondly, chronic psychological stress and friction stimulation create an “atrocious climate” for melanocyte survival. Thirdly, abnormal local immunity and thick stratum corneum result in an “aberrant soil” for melanocyte growth and drug effect. In aggregate, these factors may individually or collectively impede melanocyte survival and hinder melanin production, ultimately contributing to refractory vitiligo. Various medicines were used in individuals suffering from refractory vitiligo, yet they often showed poor effects. The chronic disease duration and poor therapeutic effects can result in persistent depression and low QoL in refractory vitiligo patients.

Refractory vitiligo is a puzzle in vitiligo management. Some therapeutic attempts have shown decent effects and have been widely adopted in many countries. In this review, we aim to elaborate on currently available treatment options in the treatment of refractory vitiligo and potential therapeutic strategies.

## 2 JAK inhibitor therapy

Previous study has confirmed that Interferon (IFN)-γ and CD8^+^ T cells play a central role in the pathophysiology of vitiligo (20). IFN-γ can activate the JAK and signal transducer and activator of transcription 1 (STAT1) signaling pathway by binding with its receptor, and eventually result in further recruitment of CD8^+^ T cells to skin lesions and melanocyte destruction (21). This process can be inhibited using JAK inhibitors such as ruxolitinib and tofacitinib (22, 23). Therefore, JAK inhibitors have recently been extensively evaluated in multiple phase II and III clinical trials, demonstrating remarkable efficacy in vitiligo management (11, 24).

Recently, several JAK inhibitors appear to be effective in patients with refractory vitiligo. As a first-generation JAK1/3 inhibitor, oral tofacitinib in combination with NB-UVB and topical calcineurin inhibitors therapy can achieve significant Vitiligo Area Severity Index (VASI) improvements on the acral regions, trunk, and extremities after 16-week treatment compared with the control group (25). Baricitinib, a newer JAK1/2 inhibitor, was reported to provide satisfactory re-pigmentation and good tolerance for patients with generalized refractory vitiligo (26). Baricitinib has also been found to promote tyrosinase activity, melanin content, and tyrosinase, tyrosinase-related protein-1 gene expression of melanocytes damaged model *in vitro* (27). As an oral selective JAK1 inhibitor, upadacitinib monotherapy caused an average improvement of 38.65% in VASI for 12 refractory patients and ameliorated their quality of life (28). No serious adverse events were noted in these studies. Similar to other treatments, JAK inhibitors have a better re-pigmentation effect on the facial area, which may be due to the high density of hair follicles and chronic sun exposure (29). As non-invasive treatments, JAK inhibitors can be administered to patients with progressing vitiligo. To date, small case series and clinical studies have been reported that JAK inhibitors are relatively safe and effective target therapies for refractory vitiligo. Nevertheless, the current research is still insufficient, and further study is needed to provide more evidence to prove the efficacy of JAK inhibitors.

## 3 Surgical intervention

Insufficient local melanocyte reservoirs may be a primary obstacle for re-pigmentation in refractory vitiligo. Surgical intervention can be used to solve this problem. Dermatologists can replenish melanocytes or melanocyte stem cells for vitiligo lesions by various transplantation techniques. Generally, surgical intervention can be roughly classified into two categories, tissue grafting and cellular grafting (30). Tissue grafting has a long history of application with relatively simple operations. In recent years, cellular grafting has advanced significantly. There are continuous methodological and technical innovations in both surgical types. However, each surgical intervention has its pros and cons, and clinicians should apply them according to the individual conditions of vitiligo patients (**Table 1**).

**Table 1:** Surgical therapies for treating refractory vitiligo.

Study	Study design	*n* patients (lesions)	Mean age, year (range)	Stability disease (months)	Preparation recipient site	Treatment	Post-Treatment	Follow-up (months)	Re-pigmentation *n* (% of total)	Adverse effects at the recipient sites	Adverse effects at the donor sites
Majid and Imran (2012) (31)	CS	40 (84)	–	≥12	Dermabrasion	UTSG	NB-UVB	3-12	>90%: 70 lesions (83.3%)	Perigraft halo (15%), graft displacement (procedure on lateral side of the neck)	Hypertrophic scarring (n=2)
Majid and Imran (2017) (32)	CS	370 (554)	24.5 (14-55)	≥12	Dermabrasion	UTSG	NB-UVB or targeted UVB or diluted topical psoralens with sun exposure or topical mometasone cream	12-60	≥75%: 437 lesions (78.9%)	Perigraft halo (12.3%), hyperpigmentation (9.9%), achromic fissures (6.3%), graft curling (n=16), vitiligo reactivation (n=8)	Transient hyperpigmentation (63.5%), local infection (5.1%), hypertrophic scarring (n=2)
Bae et al. (2018) (33)	CS	208 (230)	32.7 (5-68)	≥3	Punching	0.8-mm MPG	308 nm excimer laser and topical tacrolimus ointment	3-12+	≥75%: 181 lesions (78.7%)	Color mismatch (24.8%), cobblestone appearance (18.3%), hyperpigmentation (11.3%), perilesional halo (6.1%)	None
Kim et al. (2020) (34)	RCT	50 (100) a. (50) b. (50)	24 (6-67)	≥12	Punching	0.5-mm MPG a. Epidermal orientations b. Dermal orientations	308 nm excimer laser and topical tacrolimus ointment	3	a. ≥75%: 36 lesions (72%) b. ≥75%: 38 lesions (76%)	a. Cobblestone appearance (4%), color mismatch (2%) b. Cobblestone appearance (2%), color mismatch (2%)	–
Baweja and Chand (2020) (35)	Comparative study	35 (70) a. 35 (35) b. 35 (35)	32.85 (18-51)	≥6	Dermabrasion	a. SBEG b. ABEM	Topical/Systemic PUVASOL	12	a. >75%: 28 lesions (80%) b. >75%: 29 lesions (82.9%)	a. Perigraft halo (20%), color mismatch (20%), infection (8.6%), graft stuck on appearance (5.7%) b. Perigraft halo (14.3%), infection (5.7%), graft stuck on appearance (5.7%)	a. Hyperpigmentation (31.4%), scarring (11.4%), depigmentation (5.7%) b. None
Gao et al. (2022) (36)	Comparative study	75 (118) a. (62) b. (56)	34.17 (7-70) a. 36.69 b. 31.38	≥12	Er: YAG laser	a. SBEG b. ABEM	Topical tacrolimus ointment and some accepted excimer laser and/or NB-UVB (a: 64%, b: 55%)	12	a. ≥75%: 47 lesions (75.8%) b. ≥75%: 22 lesions (39.3%)	None	a. Pain (high), hyperpigmentation (long), scarring (n=2) b. Pain (low), hyperpigmentation (short)
Thakur et al. (2015) (37)	CS	50 (63)	23.7 (6-65)	≥12	Slits creation	FUT	Topical corticosteroid and tacrolimus or topical PUVASOL	6	≥75%: 21 lesions (33.3%)	Intraoperative bleeding (n=1), inclusion cyst (n=1), vitiligo reactivation (n=2)	None
Mohamed Mohamed et al. (2017) (38)	Comparative study	32 a. 32 b. 32	19.1 (11-27)	≥12	a. Punching b. Slits creation	a. PG b. FUT	Excimer laser	6	a. >75%: 13 lesions (40.6%) b. >75%: 5 lesions (15.6%)	a. Cobblestone appearance (90%)	–
Mokhtar et al. (2022) (39)	Comparative study	25 a. 25 b. 25	18.56 (15-25)	≥12	a. Slits creation b. Punching	a. FUT b. MPG	NB- UVB	6	a. >75%: 15 patients (60%) b. >75%: 18 patients (72%)	a. Hair growth (n=3) b. Cobblestone appearance (n=5)	a. Hyperesthesia (n=1); infection (n=1) b. Hypopigmentation (n=1)
Budania et al. (2012) (40)	RCT	41 (54) a. 21 (28) b. 20 (26)	a. 21 (12-27) b. 21.3 (14-40)	≥12	Dermabrasion	a. NCES b. SBEG	Sun exposure	4	a. ≥75%: 25 lesions (89.3%) b. ≥75%: 22 lesions (84.6%)	–	–
Holla et al. (2013) (13)	CS	36 (80)	22.6 (16-47)	≥12	Dermabrasion	NCES	Sun exposure and oral methylcobalamin supplements	6-18	>75%: 51 lesions (63.8%)	–	–
Razmi et al. (2018) (41)	RCT	30 (84) a. 30 (42) b. 30 (42)	23.37 (10-36)	≥12	Dermabrasion	a. NCES+HFCS b. NCES	Sun exposure	4	a. ≥75%: 32 lesions (76.2%) b. ≥75%: 24 lesions (57.1%)	None	a. Hyperpigmentation (n=7) b. Hyperpigmentation (n=7)
Hamza et al. (2019) (42)	RCT	20 a. 10 b. 10	a. 27 (15-45) b. 39 (14-52)	≥6	Dermabrasion	a. HFCS b. NCES	NB- UVB	3	a. ≥75%: 3 patients (30%) b. ≥75%: 2 patients (20%)	–	b. Hyperpigmentation (40%), mild scarring (20%)
Awasti et al. (2019) (43)	RCT	30 (42) a. 15 (22) b. 15 (20)	a. 24.87 b. 24.6	≥12	Dermabrasion	NCES (a. Cold trypsinization b. Warm trypsinization)	–	4	a. >75%: 20 lesions (90.9%) b. >75%: 16 lesions (80%)	–	–
Mrigpuri et al. (2019) (44)	RCT	30 (82) a. 30 (41) b. 30 (41)	24.23 (13-36)	≥12	Dermabrasion	NCES (a. 4C method b. Lab-NCES)	Sun exposure	4	a. ≥75%: 28 lesions (68.2%) b. ≥75%: 29 lesions (70.7%)	a. Infection (n=1)	None
Anbar et al. (2020) (45)	RCT	40 a. 20 b. 20	a. 36.8 (14-50) b. 28.3 (12-40)	≥6	Dermabrasion	NCES (a. Suction blister roofs b. Partial-thickness epidermal cuts)	NB-UVB	4	a. >75%: 12 patients (60%) b. >75%: 16 patients (80%)	–	a. Hyperpigmentation (100%, return to the normal color over time) b. Hyperpigmentation (90%); scarring (90%)
Dalla et al. (2020) (46)	CT	30 (90) a. 30 (30) b. 30 (30) c. 30 (30)	27.4	≥12	a. Punching b. Dermabrasion c. Dermabrasion	a. NCES b. SBEG c. MPG	None	6	a. >75%: 17 patients (56.7%) b. >75%: 18 patients (60%) c. >75%: 7 patients (23.3%)	c. Cobblestone appearance (n=2)	none
Gunaabalaji et al. (2020) (47)	RCT	20 (40) a. 20 (20) b. 20 (20)	23.9 (18-47)	≥12	Dermabrasion	a. NCES b. HFCS	Topical tacrolimus ointment and sun exposure	9	a. >75%: 14 patients (70%) b. >75%: 10 patients (50%)	a. Discharge (n=2) b. Discharge (n=2)	b. Pain (n=3)
Thakur et al. (2020) (48)	Comparative study	30 a. 15 b. 15	a. 24.9 b. 22.7	≥12	Dermabrasion	HFCS (a. FUE b. PHF)	–	4	a. >75%: 3 patients (20%) b. >75%: 0 patients (0%)	None	None
Hong et al. (2011) (49)	CT	102 a. 35 b. 67	24.2 (9-55) a. 22.7 b. 24.9	≥6	Ultrapulse CO2 laser	CMT (a. DR ratio ≤1: 10 b. DR ratio >1: 10)	–	6	a. ≥90%: 18 patients (51.4%) b. ≥90%: 38 patients (56.7%)	–	–
Verma et al. (2015) (50)	Comparative study	27 (54) a. 27 (27) b. 27 (27)	26.1	≥6	Dermabrasion	a. CMT b. NCES	–	3-6	a. >75%: 19 lesions (70.37%) b. >75%: 2 lesions (7.4%)	–	–

ABEM, automated blister epidermal micrograft; CMT; cultured melanocytes transplantation; CS, case series; CT, clinical trial; Er: YAG, Erbium: Yttrium–aluminium-garnet; FUT, follicular unit transplantation; FUE, follicular unit extraction; HFCS, non-cultured hair follicle cell suspension; Lab-NCES, laboratory-based non-cultured epidermal cell suspension method; MPG, micropunch grafting; NB-UVB, narrow band ultraviolet B; NCES, non-cultured epidermal cell suspension; PG, punch grafting; PHF, plucking of anagen hair follicles; PUVASOL, psoralen ultraviolet A solution; RCT, randomised controlled trial; SBEG, suction blister epidermal grafting; UTSG, ultrathin skin grafting; 4C, four-compartment; -, not mentioned.

### 3.1 Tissue grafting

#### 3.1.1 Thin skin grafting

The earliest reported surgical intervention is thin skin grafting. After the administration of local anesthetics, skin grafts of a certain thickness could be harvested using manual or motorized dermatomes. Ultrathin skin grafting has been the main method in thin skin grafting with grafts composed entirely of epidermis. A retrospective analysis of 370 vitiligo patients has shown that 78.9% of lesions demonstrated excellent response after ultrathin skin grafting, and >98% pigmentation could be maintained for 4 years (32). However, thin skin grafting requires specialized surgical techniques, and the obtained grafts are irregular in shape and cannot be fully utilized. The common side effects of thin skin grafting are a “perigraft halo” of depigmentation in the recipient site and hypertrophic scarring in the donor site (31). Due to greater trauma and lower utilization, thin skin grafting has become less commonly used nowadays.

#### 3.1.2 Punch grafting

PG is a cost-effective and easy-implementation method, which enables operation in an outpatient setting. The simplest method is to transfer 1-1.5 mm superficial grafts directly from donor to recipient sites. A randomized controlled trial has shown that superficial grafts had pigmentation effects similar to deep grafts with fewer side effects (51). Due to the small size of grafts, punch grafting is suitable for irregular lesions. In recent years, motorized 0.8-mm micropunch grafting (MPG) can produce uniform and elaborate grafts. A retrospective study has indicated that 78.7% (181/230) lesions achieved ≥75% re-pigmentation after a median of 6 months (33). Following that, one study has demonstrated that motorized 0.5-mm micropunch grafting could be used irrespective of the graft orientation (34). Micro-punch technologies are convenient with reduced appearance of cobblestone. However, the British Association of Dermatologists guidelines have indicated that there is insufficient evidence to recommend punch grafting in vitiligo (52). This is possible because punch grafting has seldom clinical trials in larger cohorts and relatively high evidence of cobblestone appearance.

#### 3.1.3 Suction blister grafting

Suction blister grafting (SBEG) is performed using suction devices to create local negative pressure and produce blisters. Subsequently, the roots of blisters will be collected and transplanted to the recipient area after dermabrasion (53, 54). SBEG has the advantage of low cost and no scar formation with good cosmetic results. SBEG is time-consuming and painful, while automated blister epidermal micrograft (ABEM) can alleviate these shortcomings (35). Similarly, SBEG is not appropriate for large-sized lesions, as the donor-recipient size ratio (DR ratio) is 1:1. Although ABEM is expensive, it is relatively convenient and provides a larger transplanting area. In a comparative study involving 75 patients, SBEG had better re-pigmentation than ABEM, while ABEM allowed for improved treatment satisfaction with a better operative experience (36).

#### 3.1.4 Follicular unit transplantation

Follicular unit transplantation (FUT) is characterized by the ability to transplant hair follicles unit with undifferentiated melanocyte stem cell reservoirs (37). After anesthesia, the conventional method uses a 1-mm biopsy punch or surgical removal of rectangular fragment skin to get hair follicles from the occipital region and then transplants them to the recipient areas. FUT is a labor-intensive, safe, and effective procedure with insignificant scarring. Analogous eyelash transplantation can be used to treat vitiligo-associated eyelash leucotrichia (55). Recently, minimally invasive transplantation and cosmetic technology of FUT have been developed. Using a trichiasis electrolyzer, depigmented hair follicles could be damaged and removed before transplanting to reduce depigmented hair regeneration (56). One comparative study on MPG and FUT has found that FUT had a relatively slow process of re-pigmentation, potentially because melanocytes took a long time to migrate from the hair follicle to the epidermis (39). One point to note is that FUT usually results in undesired terminal hair growth. According to a series of case reports, some methods of FUT can induce re-pigmentation while limiting additional hair growth at the same time. First, abdominal vellus hair punch grafts could be a treatment option for preventing undesired hair growth (57). Moreover, non-intact hair bulb follicle transplantation may cause less hairy and have higher cosmetic efficacy than intact hair bulbs (58).

### 3.2 Cellular grafting

#### 3.2.1 Non-cultured epidermal cell suspension transplantation

In recent years, cellular grafting has advanced rapidly. Non-cultured epidermal cell suspension (NCES) transplantation is the most common technique of cellular grafting (59). The skin used for the suspension can be prepared from partial-thickness epidermal cuts or suction blister roof grafts (45). After that, cell suspensions are extracted by laboratory methods and transplanted into de-epithelialized recipient sites. With good hair re-pigmentation, the DR ratio of NCES transplantation can be as high as 1:5-1:10 (60). In a randomized controlled study involving 41 patients with stable vitiligo, NCES has been reported to be significantly superior to SBEG in terms of re-pigmentation at 16 weeks post-surgery (40). On the other hand, NCES preparation requires a complicated laboratory setup, some studies have concentrated on streamlining processes and improving treatment efficacy. The quality of cell suspension preparation is significantly related to treatment outcome. Cold trypsinization outperforms warm trypsinization in the preparation of cell suspension while providing a higher yield of viable melanocytes (43). The four-compartment (4C) method for epidermal cell suspension preparation has been shown to reduce costs and simplify conventional NCES preparation (44). What calls for special attention is that the donor-to-recipient expansion ratios should be considered in clinical practice. A recent systematic review showed that higher expansion ratios had a close relationship with lower re-pigmentation percentages (61).

#### 3.2.2 Non-cultured hair follicle cell suspension transplantation

Hair follicle cell suspension (HFCS) collects follicular units and has comparable effective re-pigmentation to NCES, with fewer transplanted cells and donor area complications (42, 47). Hair follicles, especially the outer root sheath, contain plenty of melanocyte stem cells for excellent pigmentation. Therefore, dermatologists should pay attention to protecting these areas when harvesting hair follicles. Follicular unit extraction has better re-pigmentation than plucking of anagen hair follicles, which is likely attributed to better protection of the outer root sheath of hair follicles (48). Optimal re-pigmentation is dependent on the number of melanocytes and hair follicle stem cells as well as skin immune homeostasis, which indicates two directions to improve the treatment effect (62). Collagenase type 1 can release cells from the outer root sheath which may help to improve the therapeutic effect of HFCS (63). In addition, a randomized self-controlled study involving 30 vitiligo patients has found that the combination of NCES and HFCS attained superior re-pigmentation than NCES in range, speed, and color matching (41). One possible explanation is that keratinocytes in NCES produce integral growth factors and support melanocyte growth (64).

#### 3.2.3 Cultured epidermal suspension transplantation

Cultured epidermal suspension (CES) isolates melanocytes and keratinocytes from the collected skin. After secondary culture, the autologous epidermal grafts will be transplanted at the recipient lesion (65). There are two types of CES, melanocyte-keratinocyte transplantation and pure melanocyte transplantation, and both methods are complicated. Melanocyte transplantation expands melanocytes in the laboratory for several weeks before transplanting (66). The distinct advantage of CES is that the maximum DR ratio of CES can be 1: 60 with a lesion size of approximately 120 cm^2^ (49). A comparative study has shown that lesions treated with CES had better re-pigmentation than those treated with NCES at the 12th-week follow-up, which was probably attributed to the high density of melanocytes in CES (50). A note of caution is the choice of the donor site. It has been reported that melanocytes obtained from the face as the donor site grew fastest and had the longest total propagation time, while melanocytes from the chest and back grew the lowest (67). As a result, CES is time-consuming and expensive with harsh application conditions, which limit its application (68). Furthermore, the long-term safety of CES remains controversial. The growth factors used in melanocyte culture may increase the risk of melanoma (69).

#### 3.2.4 Jodhpur technique

Jodhpur technique (JT) is another autologous non-cultured melanocyte plus keratinocyte grafting method. JT has a simple operation and doesn’t need trypsinization. Using a dermabrader micromotor and antibiotic ointment, upper dermal cells are collected, and then the paste-like material is spread over the dermabraded recipient site (70). JT combines the donor and recipient sites by harvesting perilesional pigmented skin, avoiding two sites of superficial wounds (71). For locations lacking the above transplant technology and specialized facilities, JT is relatively simplified and cost-effective. It should be noted that JT should be strictly used for small and stable vitiligo lesions. Since large lesions mean the need for large perilesional donor sites, using JT may cause greater trauma and a higher risk of infection. Moreover, the density of transplanted melanocytes is indistinct to evaluate in JT, which makes the treatment effect uncertain.

### 3.3 Precautions for selecting surgical methods and factors affecting curative effect

#### 3.3.1 Consideration of the condition of vitiligo patients

In selecting a proper treatment, the appropriate procedure is suggested to assess the basic condition and the progression of vitiligo. First, active autoimmunity is not conducive to the survival of transplanted melanocytes. Surgical methods will have a poor effect on progressive vitiligo patients and even aggravate the condition of vitiligo. Therefore, disease stability should be evaluated before considering surgical interventions based on the scoring system, such as Vitiligo Disease Activity Score (VIDA) and Vitiligo Signs of Activity Score (VSAS) (72, 73). Surgical treatments generally have the risk of the Koebner phenomenon. For patients with stable vitiligo for at least 1 year, the Koebner phenomenon can be reduced or even prevented. Second, the size of vitiligo lesions is another point to be considered when choosing appropriate methods. Generally, tissue grafting is more suitable for small skin lesions, and cellular grafting is preferred for large lesions. Moreover, dermatologists need to consider medical and patients’ conditions, such as the medical system and circumstances of the hospital, the financial situation, and the receptivity of vitiligo patients. Finally, both dermatologists and refractory vitiligo patients are supposed to have rational expectations about surgical treatments. Young ages, segmental vitiligo, and non-acral areas are reported to be associated with better treatment outcomes (30). It should be noted that surgical interventions are not once and for all, and it is difficult to get complete re-pigmentation after a single treatment. Many factors may affect the survival of transplanted melanocytes, such as individual differences, surgical technique, and postoperative treatment.

#### 3.3.2 Comparison of different surgical methods

Surgical interventions seem to be a good treatment choice for patients with stable and refractory vitiligo. Various surgical methods bring “seed-like” melanocytes to lesions with damaged or absent melanocytes. Compared with tissue grafting, cellular grafting has become more popular due to the higher DR ratio and better therapeutic effect. Additionally, cellular grafting has fewer deficiencies such as cobblestone appearance, which is described as an important advantage (74). Thin skin grafting requires professional skill to harvest eligible grafts, and inefficient graft collection limits its therapeutic application (75). There is no significant difference between MPG and FUT in the re-pigmentation effect (12). FUT has a longer re-pigmentation time but fewer side effects, making it suitable for exposed skin (38). NCES transplantation is a more appropriate and safe method, outperforming MPG in terms of re-pigmentation rate and color matching (46). HFCS can provide a higher density of melanocytes and hair follicle stem cells, but no significant difference compared to NCES in terms of efficacy (76). The combination of HFCS and NCES has been identified to achieve better re-pigmentation (41). Meanwhile, If the final effect of cellular grafting has a little deficiency, MPG can be an adjuvant and supplement therapy (77).

#### 3.3.3 Postoperative adjunctive therapy for improving efficacy

Surgical methods selection and individual differences should be considered when treating refractory vitiligo. Furthermore, post-surgical dressings and recipient-site preparation can have a significant impact on graft survival and re-pigmentation outcomes (78). Moreover, to prevent graft loss, the graft sites should be protected for at least two weeks after surgical treatments. Phototherapy can improve surgical outcomes in vitiligo by stimulating melanocyte proliferation, inhibiting T lymphocytes, and suppressing cytokines (79). Therefore, phototherapy represented by NB-UVB is recommended as the standard phototherapy after melanocyte transplantation (80). The NB-UVB therapy was initiated 1 week post-surgery, with a frequency of twice a week for 6 months. Patients were treated initially with a 0.2 J/cm^2^ dose followed by 20% increments if tolerated (12). Other adjuvant therapies such as oral cyclosporine (3 mg·kg^-1^·d^-1^ orally for the first 3 weeks followed by 1.5 mg·kg^-1^·d^-1^ for the subsequent 6 weeks), mometasone ointment (0.1% cream twice daily for 30 days), microneedle (after topical anesthesia, received 1.5- to 2-mm needle length-assisted microneedling until pinpoint bleeding was achieved. This was done after surgical treatment 1st and 2nd months), and platelet-rich plasma (PRP) (intradermal injection of 0.1 mL of PRP using insulin syringe into each point of the transplanted graft which are placed 5 mm apart within the recipient patch. This was done at the time of the surgical procedure and monthly for the next 3 months) have been reported to result in earlier re-pigmentation (81–84). Fractional carbon dioxide (CO_2_) laser may reduce cobblestone appearance post-tissue transplantation (38, 85). More randomized clinical trials are required to determine the effects of these adjuvant therapies.

## 4 Transcutaneous drug delivery and re-pigmentation by fractional CO_2_ laser

For refractory vitiligo in the acral and bony prominent sites, the dilemma lies in the thick stratum corneum and low melanocyte density. In addition, these areas are vulnerable to chronic friction which may result in persistent abnormal immune (86). Recently, multiple clinical trials have demonstrated the effectiveness and safety of fractional CO_2_ laser-combined therapy. Due to the penetrating and restorative functions of fractional laser, laser-assisted drug delivery has become a feasible method for refractory vitiligo (**Table 2**).

**Table 2:** Fractional CO_2_ laser combination therapies for treating refractory vitiligo.

Study	Study design	*n* patients (lesions)	Mean age, year (range)	Stability disease (months)	Fr: CO_2_ laser	Period	Fr: CO_2_ Laser combination treatment	Control treatment	Results	Adverse effects of fractional laser treatments*
Liu et al. (2019) (16)	RCT	126	31 (27-41)	≥6	a. 10600 nm	Monthly, ×5 months	TBS, NB-UVB	b. TBC, NB-UVB	Significant re-pigmentation in Fr: CO_2_ laser group (P <0.05)	Pain, burning sensation, erythema, edema
Yuan et al. (87)	CT	20 (28)	(21-61)	≥12	a. 10600 nm d. 10600 nm	Monthly, ×6 months	TBS, NB-UVB	b. 1565 nm fractional laser, TBS, NB-UVB c. NB-UVB	Significant re-pigmentation in 10600 nm Fr: CO_2_ laser group (P <0.05)	Pain, burning sensation
Wen et al. (2019) (88)	RCT	21 (42)	–	≥12	a. 10600 nm	Monthly, ×3 months	Topical tacrolimus ointment, 308 nm excimer lamp	b. Topical tacrolimus ointment, 308 nm excimer lamp	No significant difference between groups	Pain, burning sensation, erythema, edema, crusting, erythema, peri-lesional hyperpigmentation
Chen et al. (2018) (89)	RCT	45 (145)	a. 25 b. 28.1	–	a. 10600 nm	Monthly, ×6 months	Topical tacrolimus cream	b. Topical tacrolimus cream	Significant re-pigmentation in Fr: CO_2_ laser group (P <0.05)	Isomorphic responses (n=3), scarring (n=1)
Li et al. (2015) (90)	RCT	25 (50)	(21–63)	≥12	a. 10600 nm	Twice a month, ×6 months	TBS, NB-UVB	b. 10600 nm Fr: CO_2_ laser, NB-UVB	Significant re-pigmentation in Fr: CO_2_ laser group (P <0.05)	Pain, burning sensation, erythema, edema
Weshahy et al. (2022) (91)	CS	30	36.3 (18-63)	–	a. 10600 nm	Triweekly, ×4 sessions	Topical 5- FU cream	–	Significant re-pigmentation after treatment (P <0.05)	Burning sensation (n=4)
Kanokr-ungsee et al. (2022) (92)	RCT	15	36	≥12	a. 10600 nm	Monthly, ×3 months	NB-UVB, topical bimatoprost	b. 10600 nm Fr: CO_2_ laser, NB-UVB, placebo	Significant re-pigmentation in Fr: CO_2_ laser group (P <0.05). No significant re-pigmentation difference between groups	Pain, burning, erythema, crusting
Kadry et al. (2018) (93)	RCT	30	32.03 (18-59)	≥12	a. 10600 nm	Biweekly, ×3 months	PRP	b. PRP c. 10600 nm Fr: CO_2_ laser d. BC	Significant improvement in both a and b groups (P <0.05). No significant difference between a and b groups	Pain (a: 23.3%, c: 33.3%), hyperpigmentation (a: 6.67%, c: 26.7%)
Afify et al. (2021) (94)	RCT	20	46.5 (19-65)	≥6	a. 10600 nm	Biweekly, ×4 sessions	PRP, NB-UVB	b. 10600 nm Fr: CO_2_ laser c. PRP d. 10600 nm Fr: CO_2_ laser, PRP e. 10600 nm Fr: CO_2_ laser, NB-UVB f. BC	Significant percentages of surface area reduction in the triple therapy group (P <0.05). No significant difference between groups	Pain, itching, expansion, erythema, crusting (n=2)
Shin et al. (2012) (95)	RCT	10	59.5 (37-74)	≥12	a. 10600 nm	Bimonthly, ×2 sessions	NB-UVB	b. NB-UVB	Significant re-pigmentation in Fr: CO_2_ laser therapy (P <0.05)	Pain, burning, erythema, and oedema
Cunha et al. (2017) (96)	RCT	4	32.25 (21-47)	≥6	a. 10600 nm	Once every four weeks, ×5 sessions	TBS, SA	b. TBS, SA	No significant difference between groups	Pain, itching (n=1)
Feily et al. (2016) (97)	RCT	20	30.6	–	a. 10600 nm	1 session	FUT, NB-UVB, topical clobetasol solution	FUT, NB-UVB, topical clobetasol solution	Significant re-pigmentation in Fr: CO_2_ laser group (P <0.05)	Tenderness, erythema
Vachira-mon et al. (2016) (98)	RCT	26 (52)	51.2	–	a. 10600 nm	Weekly, ×10 weeks	NB-UVB, topical clobetasol propionate cream	b. NB-UVB, topical clobetasol propionate cream	Significant re-pigmentation in Fr: CO_2_ laser group (P <0.05)	Pain (96.2%), edema, oozing (n=1), crusting (n=1), tiny brown spots on nail plates (n=1)

BC, blank control; CS, case series; CT, clinical trial; Fr: CO_2_ laser, fractional CO_2_ laser; FUT, follicular unit transplantation; NB-UVB, narrow band ultraviolet B; PRP, platelet-rich plasma; RCT, randomized controlled trial; SA, salicylic acid; TBC, topical betamethasone cream; TBS, topical betamethasone solution; 5- FU, 5- fluorouracil; -, not mentioned.

*Most adverse effects of fractional laser treatments will subside within a few days.

### 4.1 Unique skin-penetrating effect of fractional CO_2_ laser

Although NB-UVB phototherapy is the first choice for treating vitiligo, the effects are not always satisfactory, especially in refractory vitiligo (99). Comparatively, the excimer laser can treat small and precise skin areas with high intensity, making it more effective than NB-UVB (100). Excimer laser based on the minimal blistering dose promotes a well-tolerated treatment effect for refractory vitiligo (101). Fractional CO_2_ laser has been introduced as a valuable “add-on” treatment modality for vitiligo (102). The most direct effect is that topical medicine can be further infiltrated through microscopic treatment zones (MTZ). MTZ are formatted by fractional photothermolysis and they can promote skin restoration (103). Various cytokines and growth factors are released during wound repair. Therefore, fractional CO_2_ laser may induce melanocyte migration during the inflammation and healing phases (104). Research has shown that fractional CO_2_ laser increases IL-4, IL-10, IL-17, and IL-23 levels, which demonstrates the restoration of the Th balance of the immune system (105). The common laser settings were as follows: pulse energy of 100 mJ, spot density ranging from 150 to 200 spots/cm^2^, and 2 passes over the assigned area. No anaesthesia was used during laser sessions, and the air-cooling device can provide pain relief during the procedure. The total number of treatments ranged from 1 to 10 sessions, with treatment intervals spanning from 1 week to 2 months. The duration of treatment varied between 2.5 and 5 months. The addition of fractional CO_2_ laser treatment has proven to be significantly advantageous for individuals with refractory vitiligo. In terms of adverse reactions, fractional CO_2_ laser usually induces short-lived side effects such as pain and burning sensations, but they will soon subside (106). Common adverse reactions include transient pain, erythema, oedema, and post-laser crust. Most symptoms alleviated within a day and post-laser crusting resolved within a week. Moreover, the fractional CO_2_ laser is not suitable for activating vitiligo due to the risk of the Koebner phenomenon.

### 4.2 Combination drugs of fractional CO_2_ laser therapy

The fractional CO_2_ laser can produce microscopic vertical canaliculi. Altering the skin barrier up to the depth of the dermis, the fractional laser allows topical medications and UV radiation to penetrate deeper (87). The combination of laser with medical treatment is effective in recalcitrant acral and bony prominent sites (107). Multiple medicines have been reported for laser-assisted combination therapies in recent years (84, 88–94, 98, 108). Specifically, tacrolimus and topical corticosteroids are both first-line treatments for vitiligo with definite curative effects (5, 109). The 0.1% tacrolimus ointment was applied twice daily for 6 months, along with 3 - 6 sessions of fractional CO_2_ laser at monthly intervals (88, 89). For topical corticosteroids, compound betamethasone dipropionate solution (2.5 mg/ml) was applied to the treated area for 2 h with gauze occlusion. The dosage was determined as 0.25 g per 1% body surface area, administered immediately after the laser treatment. The entire treatment course lasted for 5 months, with monthly intervals (16). 5-fluorouracil is a medicine for skin tumors, which may improve melanocyte migration through activation of the CXCL12/CXCR4 axis (110). Patients applied 5% 5-fluorouracil cream for 5 days consecutively following each laser treatment session. There were 4 treatment sessions, 3 weeks apart. Patients were cautioned against exposing lesions to sunlight post-session (91). Bimatoprost is a prostaglandin F2α analogue, which could improve cutaneous pigmentation by activating prostaglandin F receptor and melanosome uptake by keratinocytes (111). Bimatoprost 0.01% solution was prepared by dissolving pharmaceutical grade bimatoprost powder in an ethanol/water base. Applying one drop of each bottle over the treatment area of four-square centimeters, twice daily for 12 weeks. Conducting 3 sessions of fractional CO_2_ laser at monthly intervals (92). PRP is effective in healing and regeneration since it contains a variety of mitogenic/chemotactic growth factors (112, 113). Patient’s peripheral vein was aspirated for an 8 ml blood sample, which was then centrifuged at 1,500 rpm for 5 minutes. A 30-G needle was used for superficial intradermal microinjections (0.1 mL per injection, with a 1 cm spacing). Conducting 6 sessions of fractional CO_2_ laser with intervals of 2 weeks (93). These combinations demonstrate the broad applicability of laser-assisted treatments, which are considered relatively safe treatment options. Treatment-related adverse events, primarily attributed to fractional laser, are generally resolved without special intervention soon after they were reported. Overall, laser-assisted combination therapies promote the penetration and prolong the duration of drug action. Larger clinical trials are needed to investigate drug selection of laser-assisted treatments and improve the clinical efficacy for refractory vitiligo.

### 4.3 Effect of multiple laser-combination treatments

Fractional CO_2_ laser therapy can also enhance the efficiency of NB-UVB refractory vitiligo, and they are frequently used in conjunction with medication as triple therapies (17, 92, 94–96, 98). Similarly, the added benefit of fractional CO_2_ laser has been demonstrated in surgical therapies (84, 97). However, more is not always better. Triple combination therapy of fractional CO_2_ laser, topical tacrolimus, and 308 nm excimer lamp was not superior to double therapy (88). The failure of the treatment to achieve the expected effect may be attributed to the large molecular of tacrolimus, as well as the thin crust and tissue necrosis caused by fractional laser (114, 115). In another study, when comparing the re-pigmentation area, there was no statistically significant difference between double and triple therapies about fractional CO_2_ laser, PRP, and NB-UVB (94). Consequently, large-scale randomized controlled trials are required to determine the length, interval, and strategy of treatment of fractional laser when studying the combined efficacy of effective treatments. A longer period is needed to observe laser-combination treatment outcomes and more comprehensive methods to evaluate response.

### 4.4 Similar treatments to fractional CO_2_ laser

In general, MTZ and dermabrasion are the main advantages of fractional CO_2_ laser. The following methods may have similar beneficial effects. Fractional erbium: YAG (Er: YAG) laser is a relatively gentle laser to promote drug absorption (116). Compared with fractional CO_2_ laser, fractional Er: YAG laser produces a thinner beam profile and MTZ, which results in faster recovery. However, this feature also leads to a longer treatment period for re-pigmentation. Another common method for inducing drug delivery is microneedling. Microneedling is a simple and cost-effective therapy (117). The treatment experience of microneedling is less painful in comparison to fractional CO_2_ laser. While the standardization and precision of microneedling are relatively weaker than fractional CO_2_ laser. More clinical trials are needed to compare the different efficacy of these treatments.

## 5 Melanocyte regenerative therapies

The clinical management of refractory vitiligo is challenging. The aforementioned surgical treatment delivers melanocytes and melanocyte stem cells to lesional areas. In the lesions with thick stratum corneum, fractional laser-combination therapies improve the survival of melanocytes by ameliorating the local environment. In general, re-pigmentation in refractory vitiligo mainly depends on sufficient and available melanocytes. Being the source of melanocytes, Melanocyte stem cells are mostly located in the bulge area of hair follicles (118). The bulge area is an immune privilege site that evades the immune-killing effect. Therefore, some vitiligo patients can achieve perifollicular re-pigmentation due to the retained melanocyte stem cells. Functional melanocytes in the perilesional skin also contribute to vitiligo re-pigmentation. Moreover, studies have confirmed that stem cells that can differentiate into melanocytes are also present in the dermis of hairless vitiligo lesions (119). In general, stimulating original melanocyte stem cells and supplementing regenerative melanocyte differentiation are promising therapeutic directions for refractory vitiligo.

### 5.1 Promotion of melanocytes and melanocyte stem cell activation

Treatment for improving melanocyte regeneration represents a promising approach. However, most of these methods are still in the research phase. α-melanocyte-stimulating hormone (α-MSH) is a critical regulatory protein that promotes melanogenesis and melanocyte proliferation. As a potent α-MSH analog, afamelanotide has been reported to be an effective treatment option for vitiligo. In a randomized controlled trial, the administration of subcutaneous afamelanotide implants could increase the re-pigmentation rate and total area for vitiligo patients treated with NB-UVB (120). Mesenchymal stem cells can maintain the immune homeostasis of the local environment. They have been demonstrated to promote melanocyte proliferation and anti-apoptosis by targeting the phosphatase and tensin homolog/phosphatidylinositol 3 kinase/protein kinase B pathway (121). Adipose tissue extracellular fraction is detected to ameliorate the capability to counteract oxidative stress and activate the Wnt/β-catenin pathway *in vitro* (122). The two methods mentioned above may be helpful to improve the effect of surgical transplantation.

Moreover, research about treatment directions can also focus on the pathways of melanogenesis. Wnt signal regulation has been discovered to promote melanocyte regeneration. The Wnt/β-catenin signaling pathway can activate the transcription of melanogenesis genes such as microphthalmia-associated transcription factor (123). Microinjury caused by fractional laser has been identified to improve vitiligo pigmentation via the Wnt/β-catenin pathway (124). Furthermore, p53, transforming growth factor β, and their downstream effectors have varying roles in promoting melanogenesis (125, 126). The activation of these signal pathways appears to have positive prospects for the re-pigmentation of vitiligo.

### 5.2 Stem cell therapies for melanocyte regeneration

Stem cells have unlimited proliferation potential and they can differentiate into downstream lineages. Therefore, stem cell therapies hold great promise in replenishing melanocytes for refractory vitiligo. Embryonic stem cells are the first discovered stem cell line. Human embryonic stem cell-derived melanocytes have limited immunogenicity, which is promising to be used in cellular grafting for refractory vitiligo (127). However, the main restriction is the potential ethical issues due to allogeneic transplantation. It has been reported that mouse bone marrow mesenchymal stem cells could be successfully induced to melanocytes. In mouse tissue-engineering experiments, the induced melanocytes survived in transplanted tissues successfully and had similar biological functions to normal melanocytes (128). In autologous cellular therapy, adipose-derived stem cells have been reported to generate proliferative melanocyte precursors (129). Melanocyte precursor cells can be converted into melanocytes through the activation of the typical Wnt pathway and atypical Wnt signaling pathway (130). Induced pluripotent stem cells (iPSCs) have significant advantages. In addition to the conventional advantages of stem cells, iPSCs can be established from individual patients which indicates that they have fewer ethical issues than embryonic stem cells. A related study has demonstrated that human iPSC-derived melanocytes could be integrated into the mouse hair bulb and reconstitute pigmented hair follicles (131). Therefore, this method is promising to play long-term roles in vitiligo re-pigmentation.

## 6 Psychotherapy and adjuvant therapy

Previous research has shown that the number of vitiligo patients with depression is approximately 5 times higher than that of healthy controls (132). Various psychosocial comorbidities, such as stigmatization and sleep disturbance are also significantly increased (133). These abnormalities have a negative impact on patients’ QoL, especially in patients with refractory vitiligo. Chronic stress has been reported to induce melanocyte dysfunction by overexpressed neuropeptides, such as the substance P and neuropeptide Y (134, 135). Therefore, testing and monitoring psychological distress are recommended once vitiligo is diagnosed. Multidisciplinary treatment strategies and psychoeducation are required to address the vitiligo-associated burden of the disease (136).

To improve patients’ QoL, the following methods can be used as adjuvant therapies. Firstly, if all other treatments fail in patients with widespread vitiligo, depigmentation therapies could be an alternative option to achieve uniform skin color. Q-switched neodymium: YAG laser, bleaching creams (e.g., mono-benzyl ether of hydroquinone), trichloroacetic acid, and cryotherapy are the most common methods for depigmentation (137–141). On the other hand, cosmetic camouflage can improve the QoL of vitiligo patients, particularly in children and teenagers (142). Dihydroxyacetone, general cosmetics, and various topical camouflage agents have little impact on skin lesions (143). Furthermore, micro-pigmentation, also known as medical tattooing, could involve injecting pigment particles into lesions with needles (144). Micro-pigmentation is also an effective alternative treatment for regions with non-hair bearing and prone to friction (145). Overall, vitiligo patients need ongoing psychoeducation and professional psychotherapy when necessary. Meanwhile, the above adjuvant therapies can be appropriately considered for refractory vitiligo.

## 7 Future and perspective

Precisely, the primary treatment approaches for vitiligo revolve around coordinating immune homeostasis and promoting melanogenesis. Current research has recognized multiple cytokines and pathways implicated in the pathogenesis of vitiligo. To achieve ideal therapeutic efficacy, a treatment approach targeting multi-pathogenic pathways demonstrates promising potential. The recent successful example of JAK inhibitors in the treatment of vitiligo confirmed this review. JAK/STAT inhibitors not only block IFN-γ signaling, but also enhance Hedgehog and Wnt signaling in epidermal pigmentation, which are crucial for melanocyte migration, proliferation, and differentiation (146). The Wnt/β-catenin signaling pathway is also of great significance, as it potentially protects melanocytes against oxidative stress-induced damage, suppresses the differentiation of CD8^+^ effector T cells, and enhances the activity of regulatory T cells (147–149). For patients with active and generalized vitiligo, the administration of systemic therapies with proven curative effects and high safety profiles may be advantageous (150). In the future, targeting molecular and cellular changes associated with vitiligo will address the unmet need for treating refractory vitiligo. Before a novel single drug to cure vitiligo is developed, designing rational combination treatments based on the existing drug will become the trend.

## 8 Conclusion

Until today, treatment of vitiligo remains challenging. Refractory vitiligo management is representative of the vitiligo conundrum. Potentially effective therapeutic strategies aim to stimulate melanocyte regeneration and maintain an appropriate immune environment. Surgical interventions bring healthy melanocytes to depigmentation areas. Through the formation of microscopic treatment zones, fractional CO_2_ laser promote external medicine and phototherapy penetration. As vitiligo has serious mental health hazards, psychological testing and treatment are recommended to be included in the entire process of vitiligo. Depigmentation and camouflage treatments are meaningful for patients’ QoL improvement. Molecularly targeted therapeutics and melanocyte regeneration treatment have become the focus of interest. Although several trials have evaluated therapies for refractory vitiligo, further prospective phase III trials are sparse. Development of novel therapies is in the direction of long-acting with low side effects, to improve the durability of responses and patient compliance. Many questions regarding the progression of refractory vitiligo are still unresolved, urging us to further elucidate the pathogenesis of vitiligo and to seek additional potential treatments.

## Author contributions

XW: Visualization, Writing – original draft. WW: Writing – original draft. JC: Supervision, Writing – review & editing. CL: Funding acquisition, Supervision, Writing – review & editing. SL: Funding acquisition, Supervision, Writing – review & editing.
